# 1784. Prevalence of Community-onset Group A Streptococcal Infections across 10 Regional Midwestern Hospitals

**DOI:** 10.1093/ofid/ofad500.1613

**Published:** 2023-11-27

**Authors:** Tiffany D Kuo, Maureen K Bolon, Michael Malczynski, Chao Qi, Teresa Zembower

**Affiliations:** Northwestern Memorial Hospital, Chicago, Illinois; Northwestern University Feinberg School of Medicine, Chicago, Illinois; Northwestern Memorial Hospital, Northwestern University Feinberg School of Medicine, Chicago, Illinois; Northwestern University Feinberg School of Medicine, Northwestern Memorial Hospital, Chicago, IL; Northwestern University, Chicago, Illinois

## Abstract

**Background:**

Group A *Streptococcus* (GAS) is a bacterium that can cause infections, including strep throat, skin and soft tissue infections, and necrotizing fasciitis. As of December 2022, a global outbreak of GAS infections has been noted among the pediatric population. Northwestern Memorial HealthCare (NMHC), containing ten regional hospitals, noticed a sharp increase in community-onset GAS infections affecting both children and adults. This study examines the epidemiological and clinical features of GAS infections among patients presenting to our medical system.

**Methods:**

NMHC is an all 11-hospital system. This study focuses on the 10 regional hospitals and excludes the inpatient rehabilitation hospital. Across the health system NMHC has more than 132,000 inpatient admissions annually. All GAS infections resulted through the emergency department and inpatient locations across NMHC were extracted from the electronic medical record (EMR) between December 2022 and March 2023. Clinical and microbiological data, possible exposure source, and outcomes were determined through the EMR.

**Results:**

261 GAS infections resulted across NMHC (Figure 1). Of those 261 cases, 116(44.4%) were less than 18 years of age (Table 1). In addition, 106(40.6%) patients were hospitalized due to their GAS infections. The GAS source for hospitalized patients could not be identified for 82(77.4%) cases, while 12(11.3%) were infections secondary to injury. Thirty patients admitted to the hospital were placed in intensive care units. Ten of the hospitalized patients, age 56 and older, died. Furthermore, 9(90%) patients died due to septic shock and 1(10%) to acute respiratory failure. All deaths were attributable to GAS infections.

**Figure:**
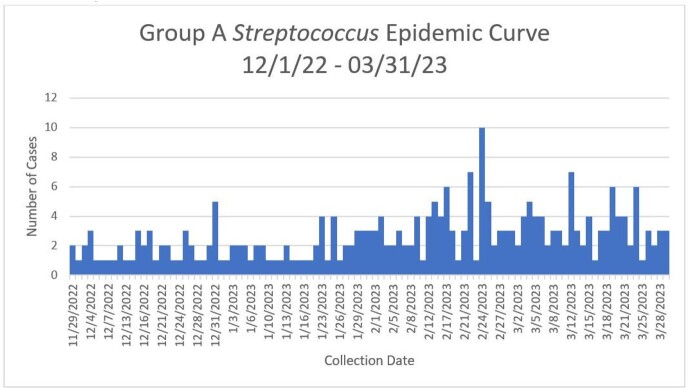

**Table 1:**
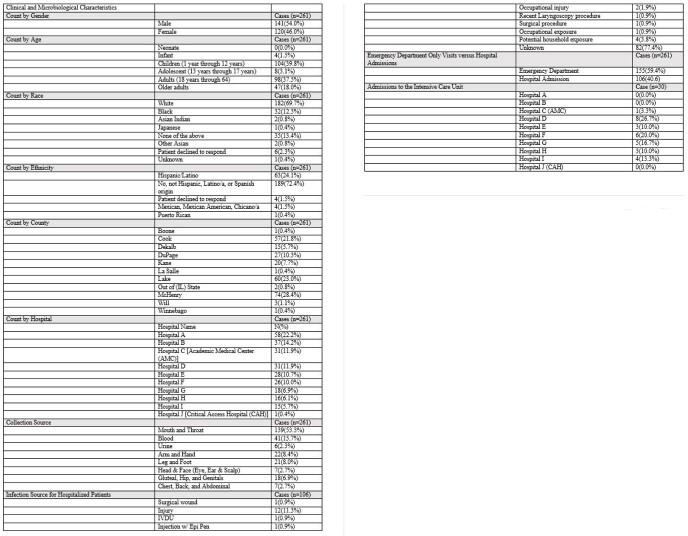
Clinical and Microbiological Characteristics

**Conclusion:**

This observation of community-onset GAS infections demonstrates a high prevalence among both pediatric and adult populations. The severity of disease and increased mortality are more predominant in the adult population.

**Disclosures:**

**All Authors**: No reported disclosures

